# A Functional Genetic Variant (N521D) in Natriuretic Peptide Receptor 3 Is Associated with Diastolic Dysfunction: The Prevalence of Asymptomatic Ventricular Dysfunction Study

**DOI:** 10.1371/journal.pone.0085708

**Published:** 2014-01-22

**Authors:** Naveen L. Pereira, Margaret M. Redfield, Christopher Scott, Nirubol Tosakulwong, Timothy M. Olson, Kent R. Bailey, Richard J. Rodeheffer, John C. Burnett

**Affiliations:** 1 Division of Cardiovascular Diseases and Department of Internal Medicine, Mayo Clinic, Rochester, Minnesota, United States of America; 2 Department of Biomedical Statistics and Informatics, Mayo Clinic, Rochester, Minnesota, United States of America; 3 Health Sciences Research, Mayo Clinic, Rochester, Minnesota, United States of America; University of Buenos Aires, Faculty of Medicine, Cardiovascular Pathophysiology Institute, Argentina

## Abstract

**Objectives:**

To evaluate the impact of a functional genetic variant in the natriuretic peptide clearance receptor, NPR3, on circulating natriuretic peptides (NPs) and myocardial structure and function in the general community.

**Background:**

NPR3 plays an important role in the clearance of NPs and through direct signaling mechanisms modulates smooth muscle cell function and cardiac fibroblast proliferation. A *NPR3* nonsynonymous single nucleotide polymorphism (SNP) rs2270915, resulting in a N521D substitution in the intracellular catalytic domain that interacts with Gi could affect receptor function. Whether this SNP is associated with alterations in NPs levels and altered cardiac structure and function is unknown.

**Methods:**

DNA samples of 1931 randomly selected residents of Olmsted County, Minnesota were genotyped. Plasma NT-proANP_1-98_, ANP_1-28_, proBNP_1-108_, NT-proBNP_1-76_, BNP_1-32_ and BNP_3-32_ levels were measured. All subjects underwent comprehensive echocardiography.

**Results:**

Genotype frequencies for rs2270915 were as follows: (A/A 60%, A/G 36%, G/G 4%). All analyses performed were for homozygotes G/G versus wild type A/A plus the heterozygotes A/G. Diastolic dysfunction was significantly more common (p = 0.007) in the homozygotes G/G (43%) than the A/A+A/G (28%) group. Multivariate regression adjusted for age, sex, body mass index and hypertension demonstrated rs2270915 to be independently associated with diastolic dysfunction (odds ratio 1.94, p = 0.03). There was no significant difference in NPs levels between the 2 groups suggesting that the clearance function of the receptor was not affected.

**Conclusions:**

A nonsynonymous *NPR3* SNP is independently associated with diastolic dysfunction and this association does not appear to be related to alterations in circulating levels of natriuretic peptides.

## Introduction

Natriuretic peptides (NPs) play an important role in maintaining intravascular volume and vascular tone by direct natriuretic, diuretic and vasodilator effects mediated by particulate guanylyl cyclase coupled receptors and modulation of the production of 3′, 5′ cyclic guanosine monophosphate (cGMP) [1]. Due to these physiological effects, the role of NPs has been well established in blood pressure homeostasis and the pathophysiology of essential hypertension [2]–[6]. Genetic variation in the NP system is associated with variation in plasma NP levels and hypertension risk [7]. Genetic variation in *NPPA*, the gene encoding the precursor of atrial natriuretic peptide (ANP) also appears to be associated with a variable response to anti-hypertensive therapy wherein C allele carriers of *NPPA* T2238C have less cardiovascular and stroke events and lower blood pressures with the use of chlorthalidone as compared to amlodipine or lisinopril [8].

In addition to its role in blood pressure regulation, the NPs play an important role in myocardial structure and function based upon cGMP mediated inhibition of cardiomyocyte growth and fibroblast proliferation [9], [10]. We have previously shown that in canines, B-type natriuretic peptide (BNP) enhances lusitropic function and coronary blood flow [11]. However, the role of metabolism and clearance of NPs and its effect on cardiac structure and function has been less extensively studied. Natriuretic peptides are degraded and cleared by membrane metallo-endopeptidase (MME) and natriuretic peptide receptor 3 (NPR3), respectively. We have demonstrated that a nonsynonymous single nucleotide polymorphism (SNP) in *MME* could result in marked reduction in *MME* expression and affect its enzymatic activity and hence its ability to metabolize NPs [12]. NPR3 is a clearance receptor responsible for removal of at least 50% of circulating NPs [13]. The gene encoding NPR3 is located on chromosome 5p13.3 and comprised of 8 exons. In a Npr3 knockout mouse model, blood pressure is lower and associated with an increased half-life of ANP, increased diuresis and reduced ability to concentrate urine [14]. NPR3 also plays an important role in K+ conductance, intracellular calcium mobilization and cellular proliferative pathways involving mitogen activated protein kinase via its interaction with Gi that could potentially affect diastolic function of the heart. The interaction with Gi is primarily mediated by a 17 amino acid activator sequence located in the middle region of the intra-cytoplasmic catalytic domain of NPR3 [15].

In a recent study, we comprehensively resequenced *NPR3* and identified 8 nonsynonymous (ns) SNPs, 7 of which were novel [15]. All except one of the nsSNPs identified had a minor allele frequency of less than 1%. The nsSNP rs2270915 identified in our study had a MAF of 12% in African-Americans, 22% in European-Americans and 17% in Han-Chinese. We have demonstrated that this SNP does not affect NPR3 protein expression however it encodes for an amino acid located in the intra-cytoplasmic 17 amino acid catalytic domain that interacts with Gi and could affect downstream signaling of the receptor [15]. This SNP has been shown to be associated with hypertension in diabetic subjects [16]. However, to date, the modulating action of this common amino acid changing NPR3 genetic variant (N521D) upon circulating NPs and myocardial structure and diastolic function remains unexplored. Thus, the goal of the current study was to investigate the impact of rs2270915 on these parameters in the “Prevalence of Left Ventricular Dysfunction Study” (PAVD). The PAVD study has significantly enhanced our understanding of the prevalence of diastolic dysfunction in the general community [17].

## Methods

The Mayo Clinic Institutional Review Board approved this study and written informed consent was obtained from all subjects.

### Study Population

The cohort consisted of a random sample of residents from Olmsted County, Minnesota, age 45 years or older who were first characterized as part of the National Institutes of Health funded “Prevalence of Left Ventricular Dysfunction Study” (PAVD) and “Cardiac Peptides in Cardiorenal Regulation” (R01 HL55502 and HL36634). The design of these prior studies and characteristics of the Olmsted County population have been previously described [17], [18]. This population has undergone comprehensive characterization by medical history, clinical examination, echocardiography and biochemical assessment that was all performed on the same day in the individual subject. Of the total 2042 subjects in the cohort, 57 subjects with heart failure or creatinine ≥2 were excluded from this analysis to avoid extremes in phenotype.

### Genotyping

Genotyping for rs2270915 was performed using the “Metabochip”, a custom Illumina iSelect genotyping array. A total of 2,112 samples were genotyped including duplicates and CEPH controls. Samples were dropped if the call rate was <98%. Samples were also dropped if there were sex errors or duplicates. The concordance rate for the duplicate samples was 99%. PLINK software was used to estimate relatedness between samples and those with a PI HAT value >0.4 reflecting twins, parent-offspring and full sibling relatedness were identified and one sample from each of those pairs were excluded. Multidimensional scaling was performed to identify non-Caucasian subjects who were then dropped from the analysis. The final sample consisted of 1931 subjects with available genotype information for the SNP of interest.

### Natriuretic Peptide Assays

Plasma NT-proANP_1-98_ and ANP_1-28_ levels were measured using a radioimmunoassay (Phoenix Pharmaceuticals, Belmont, California) [19]. A highly specific radioimmunoassay was used to measure proBNP_1-108_ as previously described [20]. The NT-proBNP_1-76_ values were measured using an electrochemiluminescence immunoassay (Roche Diagnostics, Indianapolis, Indiana) [21]. BNP_1-32_ levels were measured by Biosite Diagnostics (San Diego, California) and samples were analyzed by a fluorescence immunoassay [22]. BNP_1-32_ levels were measured by an immunoradiometric assay (nonextracted) using antibody to human BNP (Shionogi Co. Ltd., Tokyo, Japan), as previously described [23]. NPs were measured in over 90% of subjects, with some variability in numbers by specific NP.

### Doppler Echocardiography

Two-dimensional and Doppler imaging echocardiography was performed by 1 of 3 cardiac sonographers as per a standardized protocol and interpreted in a blinded fashion by a single echocardiographer (MMR). Subjects were screened for significant valvular stenosis and regurgitation. In each subject, ejection fraction was measured, and pulsed-wave Doppler examination of mitral (before and with Valsalva maneuver) and pulmonary venous inflow as well as Doppler tissue imaging of the mitral annulus was performed in each subject. Diastolic function was classified as normal (0), mild (1), moderate (2), or severe (3) on a numerical scale as previously described comprising of a composite of the ratio of mitral peak early filling velocity with velocity at atrial contraction (E/A), delta E/A with Valsalva, the ratio of peak early filling velocity with velocity of mitral annulus early diastolic motion (E/e'), mitral E velocity deceleration time, pulmonary venous systolic and diastolic forward flows, A duration and pulmonary venous atrial reversal flow [17]. Left ventricular (LV) dimension and mass and left atrial volume were calculated from M-mode and 2-dimensional measurements, respectively, and were indexed to body surface area [23], [24]. The LV mass was calculated according to the Devereux formula. Presence of LV hypertrophy was defined based on LV mass index >130 g/m^2^ for men and >100 g/m^2^ for women [25]. Presence of left atrial enlargement was defined as left atrial volume index >33 ml/m^2^ in men and >30 ml/m^2^ in women but was not used as a criteria in the composite score above for diastolic dysfunction [26].

### Statistical Analysis

Descriptive categorical variables were summarized as counts and percentages. Continuous variables that were approximately normally distributed were summarized as mean and standard deviation, non-normally distributed continuous variables were summarized by medians and interquartile ranges. These variables were transformed using the natural logarithm when evaluated in regression analyses. Initial distributions of covariates by genotype were evaluated across all 3 genotype groups. Because the G/G genotype showed a consistent difference relative to A/A and A/G genotypes, a recessive model was chosen for all analyses. Analyses were then carried out with each variable of interest modeled as the dependent variable via linear or logistic regression to test for association with the G/G genotype. Modeling was performed, both unadjusted and adjusted for age and gender. Multivariable linear and logistic regression analyses were used to evaluate whether association for natriuretic peptide levels and diastolic dysfunction respectively with rs2270915 was maintained after adjusting for other important covariates. Results of logistic regression analyses are reported as odds ratios and 95% CI. All analyses were carried out using the SAS Version 9.3 (SAS Institute Inc., Cary, NC). Two-sided tests were evaluated and p-values <0.05 were considered to be statistically significant as only one genotype was examined for this study.

## Results

### Baseline Demographics

The genotype frequencies for rs2270915 were: A/A = 1153 (59.7%), A/G = 702 (36.4%) and G/G = 76 (3.9%). Demographic data for the wild type or heterozygotes and homozygote subjects for the rs2270915 variant are outlined in **Table 1**. There were no differences in age (p = 0.26), female sex (p = 0.50) or body mass index (BMI) (age and sex adjusted p = 0.83) between the 2 groups. Renal function as measured by glomerular filtration rate (GFR) (A/A or A/G = 81 ml/min and G/G = 82 ml/min, adjusted p = 0.51) and creatinine (median 0.8 mg/dl in both groups, adjusted p = 0.49) was not statistically different. None of the subjects had clinical heart failure and the prevalence of atrial arrhythmias was low (A/A or A/G = 4% and G/G = 5%, adjusted p = 0.24) and not significantly different between the 2 groups. Genotype was not significantly associated with verified hypertension for rs2270915, however the prevalence of diabetes mellitus was only 6.8% in the entire cohort. Therefore, clinical covariates that could affect NP levels and diastolic function as described above were not significantly different between the wild type, heterozygote and homozygote subjects.

**Table 1:** Baseline Demographics of the Study Population Based on the rs2270915 Genotype.

	rs2270915		P Value	
Variable	A/A or A/G (N = 1855)	G/G (N = 76)	Unadjusted	Age/Sex Adjusted
Age at exam	62.26±10.32	63.62±10.38	0.26	
Gender, n (%)	976 (53%)	37 (49%)	0.50	
Height of patient	168.12±9.86	169.26±9.38	0.32	0.27
Weight of patient	80.60±17.89	81.82±19.07	0.56	0.60
BMI of patient	28.39±5.22	28.50±6.18	0.86	0.83
BSA	1.90±0.23	1.92±0.23	0.47	0.49
Clinical+Echo normal, n (%)	738 (40%)	25 (33%)	0.23	0.45
Diabetes, n (%)	128 (7%)	3 (4%)	0.32	0.25
CAD, n (%)	192 (10%)	13 (17%)	0.06	0.20
Verified hypertension, n (%)	500 (27%)	24 (32%)	0.37	0.51
Afib/Flutter, n (%)	68 (4%)	4 (5%)	0.47	0.73
Total cholesterol	203.59±35.64	201.58±34.44	0.63	0.74
HDL cholesterol	45.93±14.31	47.61±15.45	0.32	0.16
Systolic blood pressure	132.36±21.16	135.55±21.93	0.20	0.36
Diastolic blood pressure	73.57±10.30	73.72±9.47	0.90	0.92
Heart rate	66.17±11.34	66.36±11.26	0.89	0.88
Creatinine, median (Q1, Q3)	0.8 (0.7, 1.0)	0.8 (0.7, 0.9)	0.81	0.49
Calculated GFR (MDRD)	81.32±17.70	81.99±17.15	0.75	0.51

### Genotype and Echocardiographic Variables

NPR3 has been shown to mediate important anti-proliferative effects of the NPs [9] and hence genetic variation of the receptor could have an effect on cardiac structure and function. We therefore examined the association of rs2270915 on echocardiographic structure and function of the heart as outlined in **Table 2**. After adjustment for age and sex, rs2270915 was significantly associated with diastolic dysfunction (unadjusted p = 0.007, adjusted p = 0.02), present in 43% of homozygotes for the G allele (G/G) as compared to 28% of wild type or heterozygote subjects. The minor allele was also significantly associated with increasing severity of diastolic dysfunction (unadjusted p = 0.006, adjusted p = 0.01). Deceleration time was significantly prolonged (adjusted for age and sex p = 0.01) in the homozygote group (242 msec) compared to the wild type or heterozygote subjects (229 msec), reflecting the greater proportion of patients with mild or grade 1 diastolic dysfunction in the homozygote group. Deceleration time is typically prolonged in grade 1 diastolic dysfunction and shortens in grades 2 and 3 diastolic dysfunction. There were greater proportion of subjects in the homozygote group (52%) in whom the velocity of mitral annulus early diastolic motion or e' was 0.07 m/sec or lower and with a E/A ratio <1 (41%) than the combined group (n%, e' = 40% and E/A = 35%). In multivariable logistic regression analysis (**Table 3**) using age, sex, the presence of hypertension and body mass index as covariates, rs2270915 remained as a statistically significant independent predictor of diastolic dysfunction with an odds ratio of 1.94 (1.07–3.51, p = 0.03), the odds ratio being similar to that of hypertension (1.96, p<0.0001).

**Table 2:** Echocardiographic Parameters in the Study Population Across the rs2270915 Genotype.

	rs2270915		P Value	
Variable	A/A or A/G (N = 1855)	G/G (N = 76)	Unadjusted	Age/Sex Adjusted
Diastolic dysfunction, n (%)			0.006	0.01
None	1216 (72%)	37 (57%)		
Mild	321 (19%)	18 (28%)		
Moderate	141 (8%)	9 (14%)		
Severe	6 (0%)	1 (2%)		
Diastolic dysfunction, n (%)	468 (28%)	28 (43%)	0.007	0.02
Ejection fraction	63.18±6.47	63.20±5.98	0.98	0.84
LV hypertrophy, n (%)	161 (11%)	8 (13%)	0.65	0.87
Deceleration time (msec)	229.24±38.61	242.16±49.84	0.005	0.01
PA systolic pressure (mmHG)	22.46±4.78	23.60±5.89	0.07	0.11
LA volume index (mL/m^2^)	24.42±7.66	25.68±7.59	0.19	0.36
e' (m/sec)	0.08 0.04	0.08±0.03	0.59	0.79
e' group, n (%)			0.07	0.13
≤0.07 m/sec	622 (40%)	33 (52%)		
>0.07 m/sec	927 (60%)	31 (48%)		
E/A ratio	1.10±0.38	1.13±0.51	0.49	0.24
E/A group, n (%)			0.58	0.48
<1	642 (35%)	30 (41%)		
1–1.5	899 (50%)	32 (44%)		
>1.5	271 (15%)	11 (15%)		
Average LV mass index (g/m^2^)	96.04±21.38	98.14±21.50	0.45	0.97
LVESD index (cm/m^2^)	1.57±0.25	1.57±0.24	0.95	0.91
LVEDD index (cm/m^2^)	2.62±0.29	2.65±0.30	0.48	0.44

**Table 3:** Multivariable Logistic Regression Model for Diastolic Dysfunction (AUC = 0.828).

Variable	OR	LCL	UCL	P Value
Age	1.14	1.12	1.15	<.0001
Female sex	0.72	0.56	0.92	0.009
rs2270915 - GG	1.94	1.07	3.51	0.03
Hypertension	1.96	1.51	2.56	<.0001
Body Mass Index	1.05	1.02	1.08	0.0001

OR: odds ratio; LCL: lower 95% confidence limit; UCL: upper 95% confidence limit.

### Genotype and Circulating Natriuretic Peptide Levels

The median NP levels based on genotype for the NPR3 variant rs2270915 are outlined in **Table 4**. There was no significant difference in plasma ANP (adjusted p = 0.47) or Biosite BNP_1-32_ (adjusted p = 0.14) and Shionogi BNP_1-32_ (adjusted p = 0.25) levels between the homozygote and heterozygote or wild type subjects. There were no differences in NT-proANP_1-98_ (adjusted p = 0.53), proBNP_1-108_ (adjusted p = 0.10) and NT-proBNP_1-76_ levels (adjusted p = 0.18). The lack of difference in NPs persisted despite adjusting for other clinical covariates in addition to age and sex such as body mass index, hypertension and diastolic dysfunction. Thus, this genetic variant in *NPR3*, previously shown by us not to affect protein expression of the receptor, does not seem to affect the clearance of NPs as demonstrated by insignificant differences in circulating levels of the natriuretic peptides. In addition, the lack of difference in the circulating NPs between the 2 groups points to the functional role of the genetic variant of *NPR3* other than the clearance of NPs that could explain the increased prevalence of diastolic dysfunction observed in the homozygote group.

**Table 4:** Circulating Natriuretic Peptide Level in the Study Population According to the rs2270915 Genotype.

	rs2270915		P Value		
Variable	A/A or A/G Median (Q1,Q3)	G/G Median (Q1,Q3)	Unadjusted	Age/Sex Adjusted	MV Adjusted*
BNP (Roche NT-proBNP)	65.0 (26.8, 137.0) N = 1815	70.7 (33.6, 133.8) N = 71	0.37	0.18	0.48
Pro BNP	20.0 (10.0, 40.0) N = 1750	24.0 (9.0, 61.0) N = 69	0.11	0.10	0.27
BNP (Biosite Triage)	23.9 (9.9, 53.4) N = 1696	28.0 (14.7, 73.3) N = 69	0.13	0.14	0.37
BNP 3–32 (Shionogi)	14.4 (5.5, 30.5) N = 1854	16.1 (7.2, 37.0) N = 76	0.23	0.25	0.73
ANP	11.6 (7.5, 16.2) N = 1743	10.6 (7.4, 15.2) N = 72	0.32	0.47	0.38
NT-ProANP	2204.5 (1413.0, 3268.0) N = 1714	2138.0 (1264.0, 3293.0) N = 70	0.76	0.53	0.65

* Adjusted for age, sex, body mass index, hypertension, diastolic dysfunction.

## Discussion

This is the first study to assess and demonstrate that an amino acid changing genetic variant in *NPR3* is functional and independently associated with diastolic dysfunction in the general community, thus highlighting the possible role of NPR3 in the pathophysiology of the disease. Most importantly, the presence of diastolic dysfunction in the homozygotes for this *NPR3* genotype was independent of age, sex, BMI and hypertension with an odds ratio of 1.9 similar to that of hypertension, a well-known risk factor for diastolic dysfunction. The observation that there were no differences in circulating NPs amongst the different genotypes together with our earlier observation that this SNP does not affect the protein expression of NPR3 indicates the absence of a functional effect on NP clearance but the likely presence of a modulating effect of this SNP on downstream signaling affecting intracellular calcium handling, K+ conductance and cell proliferation [10], [27], [28]. The significant and independent association of a *NPR3* SNP with diastolic dysfunction highlights the important role of the NP system and more specifically the role of the natriuretic peptide clearance receptor in lusitropic function of the heart.

Natriuretic peptides play an important role in the pathophysiology of hypertension due to its vasodilator actions and renal action on volume homeostasis [29]. Genetic variation in the genes encoding ANP and BNP i.e. the *NPPA-NPPB* locus has been associated with variation in NP levels and hypertension [7], [30]. Importantly, NP levels are also determined by MME and the clearance receptor NPR3 in equal measure [13]. We have previously demonstrated that genetic variation in *MME* can alter the enzymatic activity of MME with implications for degradation of NPs [12]. Genetic variation in the promoter region of *NPR3* has also been demonstrated by a candidate gene approach to be associated with alterations in ANP levels and hypertension [31]. Whether a common *NPR3* genetic variant such as rs2270915 which has biological relevance given the resultant amino acid substitution in its catalytic domain that could affect downstream signalling due its interaction with Gi, could result in alteration of NPR3 activity with subsequent effects on NP levels or be associated with diastolic dysfunction was previously unknown.

The *NPR3* SNP rs2270915 has been shown in 3,126 subjects of the Diabetes, Hypertension, Microalbuminuria or Proteinuria, Cardiovascular Events, and Ramipril (DIABHYCAR) trial to be associated with hypertension in diabetics and was validated by replication in an additional 2,452 diabetic, hypertensive patients [16]. Diabetic hypertensive patients with the rs2270915 AA genotype have a more marked decrease in systolic blood pressure with salt restriction than the G carriers (−20 mmHg vs. −3 mmHg, p = 0.006) reflecting the salt sensitive nature of hypertension in these patients. In a case control candidate gene study that was focused on NPR3 polymorphisms and performed in a Chinese Han population, the G allele of rs2270915 was significantly associated with hypertension with an odds ratio of 1.55 (95% CI = 1.08–2.22) [32]. In the current study, we did not observe an association of rs2270915 with blood pressure or hypertension. We speculate that in contrast to the study by Saulnier et al and Liu et al performed in subjects with hypertension that in our general adult population with a low prevalence of diabetes (6.8%) and hypertension (23%) this genetic variant has yet to manifest itself in terms of modulating blood pressure. This is also consistent with our previous report *in vitro* that this SNP does not affect NPR3 protein expression [15] and the present study suggesting no affect on the clearance function of the receptor as demonstrated by absence of a significant difference in NP levels in the homozygotes as compared to the wild type plus heterozygote group. Hence the association of diastolic dysfunction was not related to alteration in circulating NP levels or hypertension.

One could further speculate that the role of the *NPR3* genetic variant in diastolic function could be explained by the modulation of the receptor function that mediates the actions of CNP in activating K+ conductance and hyperpolarizing smooth muscle cells via Gi [27]. Furthermore, ANP binding to NPR3 also results in activation of phospholipase C-β via Gi mediated inhibition of adenyl cyclase leading to the formation of inositol triphosphate and phosphotidyl inositol biphosphate leading to intracellular calcium mobilization [28]. It should be noted that the rs2270915 SNP results in an amino acid substitution N521D affecting the peptide sequence of the intra-cytoplasmic domain of the receptor that interacts with Gi hence perhaps altering the ability of CNP or ANP to mediate K+ conductance or to alter intracellular calcium handling respectively and thus have an impact on myocardial diastolic function.

While the NPRC is present in multiple cell types such as cardiomyocytes, cardiac fibroblasts, endothelial cells and vascular smooth muscle cells, we speculate that the mechanism of the *NPR3* genetic variant upon modulating diastolic function most likely involves dysfunction of the cytoplasmic domain within cardiac fibroblasts contributing to cardiac fibrosis and impaired diastolic function. Indeed, a role for NPRC signaling in mediating the anti-proliferative actions of CNP has recently been reported [33]. Further, we now know that the 37 amino acid cytoplasmic domain of NPR-C contains multiple Gi activator sequences which have been shown to inhibit adenylyl cyclase activity and to attenuate Ang II-, endothelin-1 (ET-1)- and arginine-vasopressin (AVP)-induced increased proliferation of vascular smooth muscle cells via the MAP kinase and phosphatidylinositol 3-kinase (PI3K) pathways [10]. As rs2270915 SNP alters the amino acid sequence of the intra-cytoplasmic domain of the receptor, we advance the speculation that there is attenuation of the ability to activate post receptor signaling with impairment in the anti-proliferative actions of the native natriuretic peptides resulting in fibroblast proliferation and increased myocardial stiffness in the presence of this SNP. Indeed, it would be intriguing to assess the modulating action of rs2270915 upon cardiac collagen content assessed with noninvasive and/or with myocardial biopsy.

The goal of this population study was to extend our *in vitro* work of genetic variants of degradation and clearance mechanisms for the natriuretic peptides to a general community with a focus on the *NPR3* SNP rs2270915 due to its biological relevance as described above. A strength of this study is the use of a large well-characterized sample of the general population 45 years or older from Olmsted County, Minnesota with extensive measurements of the NPs, clinical phenotyping with in-depth echocardiography that has helped define diastolic dysfunction in the community. Indeed, our study serves to validate a pathophysiological phenotype of rs2270915 by demonstrating an association with diastolic impairment in the general population. A limitation is that we have used a relatively homogenous population of European ancestry thus requiring replication in another population of different ethnicity. A second limitation is that our assessment of diastolic function was non-invasive and more in-depth hemodynamic studies in such subjects would important validate the current echocardiographic derived observations.

In summary, in a random sample of the general population, the association of a functional *NPR3* nonsynonymous SNP rs2270915 was evaluated to understand the role of genetic variation of *NPR3* on regulating circulating NPs and its possible association with myocardial structure and function. The variant rs2270915, a nonsynonymous SNP in exon 8 of *NPR3*, was not associated with a difference in plasma levels of different molecular forms of ANP or BNP. However, after adjustment for age, sex, BMI and hypertension, rs2270915 was significantly associated with diastolic dysfunction. The significant association of this *NPR3* genetic variant with diastolic dysfunction was confirmed by assessing multiple echocardiographically derived Doppler indices presented as a composite score underscoring the importance of the downstream effects of NP binding with NPR3 on K+ conductance and intracellular calcium handling and/or fibroblast and smooth muscle cell proliferation. We have demonstrated for the first time the functional significance of the *NPR3* variant rs2270915 by its independent association with diastolic dysfunction. The role of NPR3 in the pathophysiology of diastolic dysfunction other than its ability to affect the clearance of natriuretic peptides needs to be further explored.
